# WY195, a New Inducible Promoter From the Rubber Powdery Mildew Pathogen, Can Be Used as an Excellent Tool for Genetic Engineering

**DOI:** 10.3389/fmicb.2020.610252

**Published:** 2020-12-21

**Authors:** Yi Wang, Chen Wang, Mamy Jayne Nelly Rajaofera, Li Zhu, Xinze Xu, Wenbo Liu, Fucong Zheng, Weiguo Miao

**Affiliations:** ^1^Key Laboratory of Green Prevention and Control of Tropical Plant Diseases and Pests (Hainan University), Ministry of Education, Haikou, China; ^2^College of Plant Protection, Hainan University, Haikou, China; ^3^Hainan Academy of Ocean and Fisheries Sciences, Haikou, China

**Keywords:** obligate biotrophic fungi, *Oidium heveae*, inducible promoter, functional verification, monocotyledons, dicotyledons, *hpaXm*, TMV resistance

## Abstract

Until now, there are few studies and reports on the use of endogenous promoters of obligate biotrophic fungi. The WY195 promoter in the genome of *Oidium heveae*, the rubber powdery mildew pathogen, was predicted using PromoterScan and its promoter function was verified by the transient expression of the β-*glucuronidase* (*GUS*) gene. WY195 drove high levels of *GUS* expression in dicotyledons and monocotyledons. qRT-PCR indicated that *GUS* expression regulated by the WY195 promoter was 17.54-fold greater than that obtained using the CaMV 35S promoter in dicotyledons (*Nicotiana tabacum*), and 5.09-fold greater than that obtained using the ACT1 promoter in monocotyledons (*Oryza sativa*). Furthermore, WY195-regulated *GUS* gene expression was induced under high-temperature and drought conditions. Soluble proteins extracted from WY195-*hpaXm* transgenic tobacco was bioactive. Defensive micro-HR induced by the transgene expression of *hpaXm* was observed on transgenic tobacco leaves. Disease resistance bioassays showed that WY195-*hpaXm* transgenic tobacco enhanced the resistance to tobacco mosaic virus (TMV). WY195 has great potential for development as a new tool for genetic engineering. Further in-depth studies will help to better understand the transcriptional regulation mechanisms and the pathogenic mechanisms of *O. heveae*.

## Introduction

Promoters regulate the expression of genes. Microorganisms and plants are the two main sources of promoters used for plant genetic engineering. To date, the most important biotechnology use of filamentous fungi is in the production of biological products, such as antibiotics, organic acids, and many commercial enzyme preparations (Brandl and Andersen, 2015). However, filamentous fungi, are lower eukaryotes, their endogenous promoters are being increasingly used to express endogenous genes or to express exogenous genes in plants or animals (Nevalainen et al., 2018). The most significant step forward in recent years has been the creation of multiple-protease-deficient expression hosts, which has resulted in gram/liter yields of proteins of human origin (Landowski et al., 2015). Filamentous fungi can express surprisingly high levels of endogenous genes, which indicates that endogenous genes are regulated by powerful promoters. This implies that filamentous fungi may be a good source of potential promoters that can be developed for biotechnological applications. There are few studies and reports on the use of endogenous promoters of filamentous fungi to express exogenous genes in filamentous fungi. Examples of inducible promoters that have been used to express exogenous genes of filamentous fungi include the *Trichoderma reesei* cbh1 promoter (Cherry and Fidantsef, 2003), the *Aspergillus* glaA promoter (Brunt, 1986), and the *Aspergillus nidulans* alcA promoter (Hintz et al., 1995); examples of constitutive promoters include the *Aspergillus nidulans* gpdA promoter (Punt et al., 1990) and the *Trichoderma* pki1 promoter (Mach et al., 1994).

Obligate biotrophic fungi cannot be cultivated *in vitro*. As a result, many of its genetic operations are difficult to achieve, research on all aspects of the genetic transformation and molecular biology of obligate biotrophic fungi are seriously lagging behind that of other filamentous fungi. *O. heveae* is the causal pathogen of powdery mildew, which is one of the most economically damaging diseases of rubber trees (*Hevea brasiliensis*), which are the main source of natural rubber. *O. heveae* is an obligate parasitic fungi that cannot be cultured *in vitro* (Tu et al., 2012), and molecular research of this pathogen is extremely backward (Liyanage et al., 2016).

Harpin proteins are ubiquitous in Gram-negative bacterial pathogens and are encoded by a hypersensitive response and pathogenicity (*hrp*) gene cluster that stimulates plants to produce a hypersensitive response (HR) class of protein elicitors. Harpin proteins not only induce a plant disease response when endogenously expressed but also induce plant disease resistance when applied externally (Ding, 2017). *HpaXm* is a *hrp* gene obtained from *Xanthomonas citri* ssp. *malvacearum* and its protein is named hpaXm (Miao et al., 2010). At present, hpaXm is classified as a harpin protein in the “others” group. When applied exogenously, hpaXm induces plants to produce a HR and enhances the resistance of tobacco to *Tobacco mosaic virus* (TMV). Li et al. (2017) transformed *hpaXm* and its signal peptide fragment HpaXmΔLP into *N Nicotiana tabacum*. Soluble protein extracted from *hpaXm* transgenic tobacco can stimulate a HR in tobacco. Endogenous expression of *hpaXm* can cause a micro-HR in tobacco, and can induce tobacco resistance to TMV (Li et al., 2017).

The aim of this study was to provide a new tool for plant genetic engineering and to promote molecular research studies of *O. heveae*. PromoterScan online software^1^ was used to predict the genome sequence of *O. heveae*, and the predicted promoter sequence WY195 was obtained. Promoter function verification and quantitative determination of expression levels showed that WY195 was a strong and efficient promoter. Furthermore, WY195 not only regulated the highly efficient expression of exogenous genes in dicotyledons but also regulated the efficient expression of exogenous genes in monocotyledons and, hence, has great potential for development. In addition, a plant expression vector that regulates the expression of *hpaXm* by WY195 was constructed and transformed into *N. tabacum* to investigate the effect on the disease resistance of transgenic plants and demonstrate the practicality of the WY195 promoter.

## Materials and Methods

### Plant and Fungal Materials

*Oidium heveae* Steinm. strain HO-73 [provided by the Key Laboratory of Green Prevention and Control of Tropical Plant Diseases and Pests (Hainan University), Ministry of Education, China] was used in this study. The pathogen was cultured on young, bronze-stage leaves of the moderately susceptible rubber tree cultivar Reyan 7-33-97 (Wan et al., 2014). The rubber plants were grown in a controlled growth chamber at 25°C with a 16 h: 8 h, light:dark photoperiod. Tobacco (*Nicotiana tabacum* L.), dragon fruit (*Hylocereus undatus* (Haworth) Britton and Rose), rice (*Oryza sativa* L. ssp. *japonica* cv. Nipponbare), barley (*Hordeum vulgare* L.), and maize (*Zea mays* L.) were all grown in a controlled growth chamber at room temperature (25–30°C) with a 16 h:8 h, light: dark photoperiod.

### Promoter Prediction

The whole genome sequence of *O. heveae* was analyzed and reported in a previous study by our laboratory (Liang et al., 2019). PromoterScan was used to predict promoters in the *O. heveae* genome (Bajić, 2000; Ioshikhes and Zhang, 2000; West et al., 2001; Zhang et al., 2008).

### Verification of WY195 Promoter Function

Based on the predicted sequence of WY195, primers were designed using Primer Premier 5.0 (Premier Biosoft International, CA, United States), and *Bam*HI and *Hin*dIII restriction sites were introduced. The primers were synthesized by the Beijing Genomics Institute. The *WY195* gene was PCR-amplified from the whole genome of *O. heveae* (using primers WY195F/WY195R).

The pBI121 vector (TIANNZ, 60908-750y) contains the kanamycin resistance gene (*KanR*) and the reporter gene β-*glucuronidase* (*GUS*). *GUS* is regulated by CaMV 35S promoter (35S). We digested the 35S promoter carried by the pBI121 vector using the restriction endonucleases *Hin*dIII [R0104S, New England Biolabs (NEB), Ipswich, MA, United States] and *Bam*HI (R0136V, NEB) to use it as a backbone. The predicted promoter WY195 was recovered after the TA clone was correctly sequenced and then introduced into the site of the original 35S promoter to construct our recombinant vector.

The recombinant vector PBI121-WY195 was transformed into *Agrobacterium tumefaciens* strain LBA4404 by triparental hybridization (Wang and Fang, 2002), and then transformed into *N. tabacum* using the *Agrobacterium*-mediated leaf disk method (Horsch et al., 1985) for transient expression. Finally, the WY195 promoter function was verified by *GUS* staining (Jefferson et al., 1987).

## Characteristics Analysis of WY195

### WY195 Expression Range

After determining that WY195 can transiently regulate the expression of *GUS* in tobacco, we investigated whether WY195 can regulate the expression of exogenous genes in other plants. To investigate the expression range of WY195 regulation, we transiently expressed the *GUS* gene in young, bronze-stage rubber tree leaves and young pitaya (dragon fruit) stalks (dicotyledons), rice callus, young barley leaves and immature maize embryos (monocotyledons) using the *Agrobacterium tumefaciens*-mediated transformation (ATMT) method.

### Validation of Relative Gene Transient Expression Using Quantitative Real-Time-PCR

The *N. tabacum* leaf disks and *O. sativa* callus in transient expression were partially used for *GUS* staining, and the remaining part was used to extract RNA and then reverse transcription. The cDNA that was generated was diluted 1,000 times as a template and real-time fluorescence quantitative PCR was performed to verify the transient expression level of *GUS*.

RNAprep Pure Plant Kit (TIANGEN, DP441) was used for total RNA isolation, the Revert Aid First Strand cDNA Synthesis Kit (Thermo Fisher Scientific, K1621) was used for cDNA synthesis, and SYBR Premix Ex Taq II (TAKARA, RR820A) was used for qPCR. qRT-PCR was performed using a QIANGEN Rotor-Gene Q MDx RealTime PCR system with the following PCR conditons: Step 1, 94°C for 30 s; step 2, 45 cycles of 94°C for 12 s, followed by 58°C for 30 s and 72°C for 30 s; step 3, 72°C for 10 min. A *-tubulin* were used to normalize mRNA levels. The α*-tubulin* primers: α-tubulinF (5′-TCTGAACCGACTTATTTCAC-3′) and α-tubulinR (5′-CATTGACATCCTTTGGCACA-3′). The *GUS* primers: GUSF (5′-GTCGCGCAAGACTGTAACCA-3′) and GUSR (5′-TGGTTAATCAGGAACTGTTG-3′). The relative expression level of the *GUS* gene was calculated using the 2^–ΔΔCt^ method (Arocho et al., 2006; Adnan et al., 2011). Data were obtained from 3 sets of experiments and analyzed using SPSS version 16.0 software (SPSS Inc., Chicago, IL, United States).

### WY195 Promoter Type

The recombinant vector pBI121-WY195 was transformed into *N. tabacum* using ATMT and stably expressed. Tissue culture was used to regenerate the explants. The T0 generation seeds were collected and the T1 generation transgenic tobacco were planted. The leaves of the T1 generation transgenic tobacco (40 days) were collected for DNA extraction. PCR and Southern blot were performed to verify whether the transformation had succeeded.

*GUS* staining was performed on the tissues or organs of transgenic tobacco plants that were successfully transformed. If the *GUS* staining result is positive, it would be determined based on the staining result that the promoter belongs to a constitutive promoter or an organ/tissue-specific promoter. If the staining result is negative, the plant *cis-*acting regulatory element database PlantCARE would be used to analyze the sequence of WY195. Then, based on this, the corresponding induction would be carried out to analyze the type of the promoter.

Temperature induction was improved (Baker et al., 1994; Éva et al., 2018) as follows: transgenic tobacco leaves were induced at 4°C for 48 h, and 42°C for 48 h. Drought induction was improved (Jang et al., 2004; Hua et al., 2006) as follows: transgenic tobacco seedlings were introduced into a centrifuge tube containing 30% PEG 6000 for hours. Light induction was improved (Xu-Jing et al., 2009; Albers and Peebles, 2017) as follows: transgenic tobacco for continuous light incubation for 72 h. Anaerobic induction was improved (Niemeyer et al., 2011; Li et al., 2016) as follows: transgenic tobacco seedlings are placed in water for 2/48 h. Wound induction was improved (Kesanakurti et al., 2012; Hernandez-Garcia and Finer, 2016): transgenic tobacco leaves for 2/48 h induction of puncturing wounds.

### WY195-Regulated Expression of *hpaXm* in Tobacco

#### TMV Resistance

The functional gene *hpaXm* which regulated by WY195 promoter was stably expressed in *N. tabacum* using ATMT. T1 generation transgenic tobacco were inoculated with TMV at 40 days after transplanting to soil. Inoculation was performed for TMV (100 μl; 18 μg/ml of solution) by rubbing leaves using a finger in the presence of abrasive diatomaceous earth (Klement et al., 1990; Dong and Beer, 2000). Inoculated tobacco were grown in a greenhouse maintained at room temperature (25–28°C) and investigated for infection 7 days later (Dong and Beer, 2000; Peng et al., 2004). The number and size of lesions on leaves were determined. Disease severity was expressed as number of lesions per leaf for TMV. Assays were conducted three times, each involving 10 plants. For all quantitative determinations, the data were analyzed by Duncan’s multiple range test at *P* ≤ 0.01. For differences in disease severity, the T1 generation of WY195-*hpaXm* transgenic tobacco and the T1 generation of 35S-*hpaXm* transgenic tobacco were all compared against *N. tabacum* wild type plants.

#### Protein Analysis and Micro-HR Observation

Soluble proteins were isolated from WY195-hpaXm transgenic tobacco leaves (Bollag et al., 1996). PMSF at 0.1 mol/L was added to protect proteins from destruction by proteases (Wei et al., 1992). The bioactivity in aqueous solutions were tested. Purified proteins were resolved with SDS-PAGE. A portion of the aqueous solution of the protein preparation was heated in a boiling water bath for 10 min while the other portion was left untreated. The biological activity of both was tested and resolved by native PAGE. Biological activity was boiled for 10 min (Schechter et al., 2004). Evaluated the tobacco HR response for it in comparision with *hpaXm* from *E. coli* strain BL21/pGEX-*hpaXm*.

Micro-HR can monitored by observing dead cells after leaves trypan blue staining (Uknes et al., 1992; Alvarez et al., 1998; Peng et al., 2003). Approximately 1 cm × 1 cm tobacco leaves were placed in lactophenol trypan blue solution, which contains 15.45% aqueous, 18.18% water saturated phenol, 17.82% glycerol, 18.18% distilled water and 27.27% trypan blue. Then the treated leaves were heated in a boiling water bath for 5–10 min and incubated at room temperature for 6–8 h, and were observed after decolorization. The protein of the pBI121-*hpaXm* with the same treatment was used as a positive control, and that of the empty vector (pBI121) transgenic tobacco and *N. tabacum* wild type were used as two different negative control. To determine if the two reactions spontaneously occurred in the transgenic lines, every three leaves on the plants were similarly studied at 10 day intervals until flowering.

## Analysis of WY195 Promoter Full Length

The 2,000 bp upstream of the WY195 transcription start site (TSS) plus the downstream 100 bp sequence was considered as the research object and was named WY195Q. The Transcription factor (TFs) of WY195Q were predicted by online database PROMO^2^ (promoinit.cgi?dirDB = TF_8.3). Then the TFBS (Transcription factor binding site) of these TFs were searched in online database JASPAR^3^. The function of these searched TFBS had been reported. And these TFBS were marked on the sequence of WY195Q. Serial deletion mutations of WY195Q were performed after avoiding the destruction of these TFBS with known functions. Specific primers were designed, and the plant expression vectors were constructed after TA cloning. Transient expression was conducted in *N. tabacum*, and the expression levels were determined by qRT-PCR. The expression levels to each other were compared to initially determine the effective full length of the promoter.

## Results

### Promoter Prediction

A TATA-box at 48,476 bp and a TSS at 48,506 bp in one gene of the *O. heveae* genome were predicted using PromoterScan (Version 1.7) (Table 1), and the predicted promoter was named WY195. There is no homologous sequence with WY195 aligned in NCBI all database. At the same time, no promoter with the same sequence of WY195 searched in the eukaryotic promoter database (EPD). This shows that WY195 is a new promoter. We applied for a GenBank accession number (MK049253).

**TABLE 1:** Promoter prediction of WY195 using PromoterScan.

**Promoter region predicted on forward strand in 48252–48502 Promoter Score: 53.25 (Promoter Cutoff = 53.000000) TATA found at 48476, Est.TSS = 48506**						
**Significant Signals**	**TFD #**	**Strand**	**Location**	**Sequence**	**Site**	**References**
junB-US2	S01738	+	48,464	GGCCAAT	junB-US2	Nucleic Acids Res 19: 775–81 (1991)
NFI	S00281	+	48,465	GCCAATC	NFI.2	Mol Cell Biol 7: 3646–55 (1987)
CTF	S00780	−	48,471	GATTGG	Hsp70.5	Mol Cell Biol 7: 3646–55 (1987)
TFIID	S00087	+	48,477	TATAAAA	Ad2MLP US.5	Cell 43: 165–75 (1985)
TFIID	S00615	+	48,477	TATAWAW	TATA-box-CS	Annu Rev Biochem 50: 349–83 (1981)
TFIID	S01540	+	48,477	TATAAA	TATA-box.2	Nucleic Acids Res 14: 10009–26 (1986)

### Verification of WY195 Promoter Function

**FIGURE 1:**
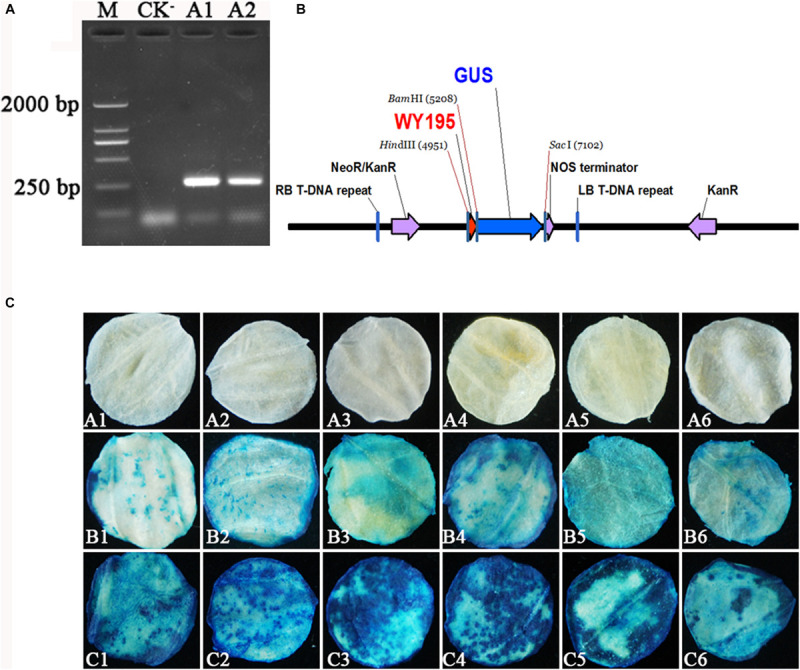
Verification of WY195 promoter function. **(A)** Amplification of WY195. (M) Marker 2000; (CK^−^) Negative control with ddH_2_O as template; (A1) and (A2) WY195. **(B)** Map of recombinant plant expression vector pBI121-WY195. **(C)** Histochemical staining for *GUS* activity in transiently transformed leaf discs of *N. tabacum*. (A1–A6) CK^−^, negative control, leaf discs of *N. tabacum* wild type; (B1–B6) CK^+^, positive control, *GUS* activity in leaf discs regulated by the 35S promoter; (C1–C6) *GUS* activity in leaf discs regulated by the WY195 promoter.

WY195 was amplified by primers WY195F/R (5′-CCCAAGCTTAACCAATAATTTTCACGAGGG-3′/5′-CGG GATCCTCTGCATGCTAGTGATTTGTT-3′), which generated a single clear PCR product of 251 bp (Figure 1A). The WY195 sequence was successfully introduced into pBI121 to construct the plant expression vector pBI121-WY195 (Figure 1B).

WY195 was transiently expressed in *N. tabacum* by performing ATMT. The WY195-regulated reporter gene *GUS* was successfully expressed in *N. tabacum*, producing β-glucuronidase, which decomposed X-Gluc to form a blue-colored substance. *N. tabacum* leaf discs expressing *GUS* regulated 35S promoter, which acted as a positive control, were also stained blue, whereas the *N. tabacum* wild type leaf discs, which acted as a negative control for *GUS* expression, were not stained blue (Figure 1C). These results demonstrate that the WY195 sequence has a promoter function and is an endogenous promoter of *O. heveae*. At the same time, we can observe that WY195 staining was significantly better than 35S promoter (deeper blue). This indicates that the ability of WY195 to regulate *GUS* expression is much higher than 35S.

## Characteristics Analysis

### Expression Range and Relative *GUS* Gene Transient Expression Levels Regulated by the WY195

**FIGURE 2:**
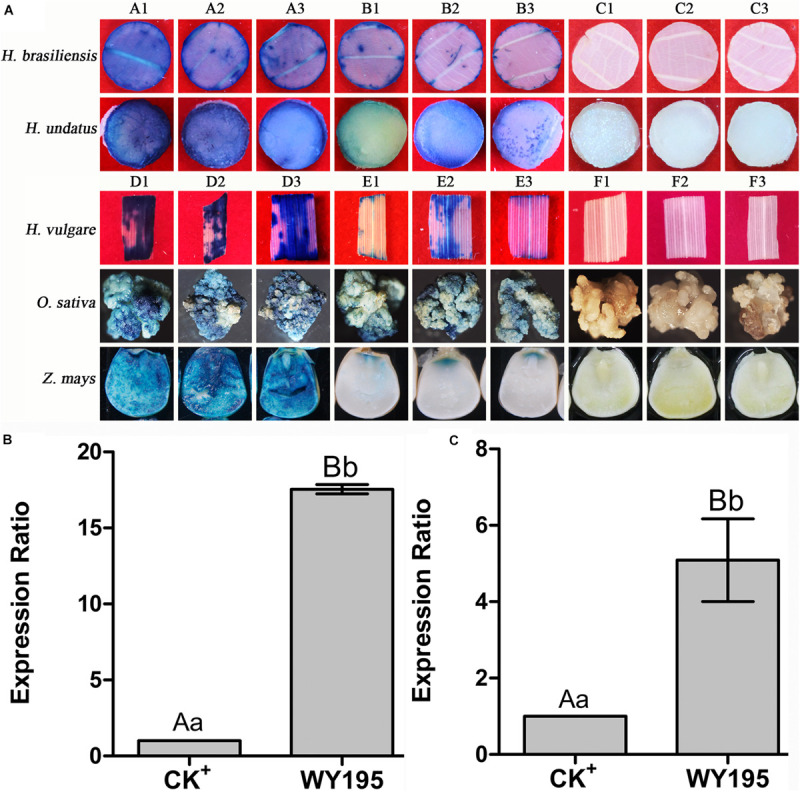
*GUS* transient expression regulated by WY195 promoter in monocotyledons and dicotyledons. **(A)** Histochemical staining for GUS activity in transient transformed dicotyledons and monocotyledons. (A1–A3) GUS activity in dicotyledons regulated by the WY195 promoter; (B1–B3) CK^+^, positive control, GUS activity regulated by the 35S promoter; (C1–C3) CK^−^, negative control, GUS activity in wild type. (D1–D3) GUS activity in monocotyledons regulated by the WY195 promoter; (E1–E3) CK^+^, positive control, GUS activity regulated by the ACT1 promoter; (F1–F3) CK^−^, negative control, GUS activity in wild-type. **(B)** Transient expression level of *GUS* regulated by WY195 promoter in dicotyledons (*N. tabacum*); CK^+^, 35S. **(C)** Transient expression level of *GUS* regulated by WY195 promoter monocotyledons (*O. sativa*); CK^+^, ACT1 promoter. Different lowercase letters indicate a significant difference (*P* < 0.05), and different uppercase letters indicate a very significant difference (*P* < 0.01).

Histochemical staining for GUS activity revealed that WY195 drove the efficient expression of the exogenous gene (*GUS*) in transiently transformed dicotyledons (tobacco, rubber, and dragon fruit) and monocotyledons (barley, rice, and maize) (Figure 2A).

qRT-PCR indicated that *GUS* expression regulated by the WY195 promoter was 17.54-fold greater (*P* ≤ 0.01; Figure 2B) than that obtained using the 35S promoter in dicotyledons (*N. tabacum*), and it was 5.09-fold greater (*P* ≤ 0.01; Figure 2C) than that obtained using the ACT1 promoter in monocotyledons (*O. sativa*).

### Promoter Type

**FIGURE 3:**
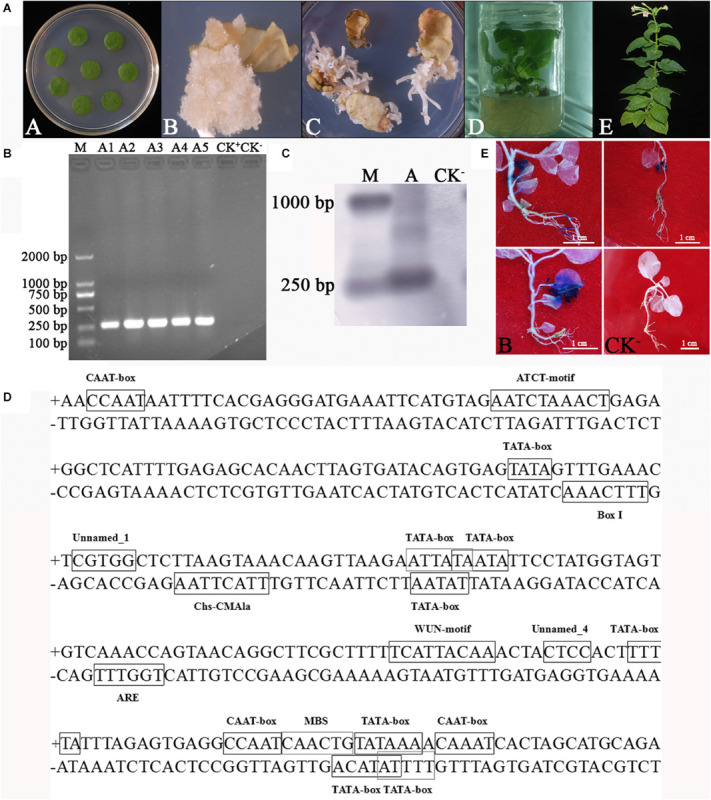
Analysis of WY195 promoter type. **(A)** Stages of *Agrobacterium*-mediated tobacco transformation. (A) Tobacco leaf disks for co-cultivation with *Agrobacterium* inoculum. (B) Callus of *N. tabacum*. (C) Adventitious buds. (D) 14-day-old plantlets that survived on Kanamycin selection media transplanted to a jar. (E) Mature plants transplanted to soil. **(B)** PCR amplification. (M) Marker 2000; (A1)-(A5) WY195-*GUS* transgenic tobacco plants; (CK^+^), pBI121 transgenic tobacco plants; (CK^−^) *N. tabacum* wild type. **(C)** Southern blot analysis of WY195 in the genome of transgenic *N. tabacum*. (M) DNA molecular weight marker (DIG-labeled); (A) WY195 promoter; (CK^−^) *N. tabacum* wild type. **(D)** Sequence analysis of predicted promoter WY195 by PlantCARE. **(E)** Histochemical staining for GUS activity in a T1 generation of WY195 transgenic tobacco grown under different conditions. (A) High temperature induced WY195-*GUS* transgenic tobacco; (B) Drought-induced WY195-*GUS* transgenic tobacco; (CK^+^) 35S-*GUS* transgenic tobacco; (CK^−^) *N. tabacum* wild type.

pBI121-WY195 (LBA4404 strain) was transformed into *N. tabacum* using the ATMT method. The transformed tobacco discs were tissue cultured, then became the T1 generation transgenic tobacco plants (Figure 3A). PCR and Southern blot demonstrated that WY195 was successfully transformed into *N. tabacum* (Figures 3B,C). However, *GUS* staining was not observed in the tissues or organs of the T1 generation of transgenic tobacco plants. Therefore, we concluded that WY195 is not a constitutive promoter or an organ/tissue-specific promoter but an inducible promoter.

PlantCARE analysis revealed that the WY195 sequence comprised nine copies of the TATA-box, five copies of the CAAT-box, a wound-responsive element, three elements related to the light response, one *cis-*acting regulatory element essential for anaerobic induction, one MYB *cis-*acting regulatory element, one binding site involved in drought-inducibility, and three *cis*-elements, the functions of which were unclear (Figure 3D and Table 2).

**TABLE 2:** Sequence analysis of WY195 promoter using PlantCARE.

***Cis-*acting regulatory elements**	**Organism**	**Position**	**Strand**	**Sequence**	**Function**
TATA-box	Arabidopsis thaliana	87	+	TATA	Core promoter element around −30 of transcription start
	Helianthus annuus	223	−	TATACA	Core promoter element around −30 of transcription start
	Glycine max	132	+	TAATA	Core promoter element around −30 of transcription start
	Lycopersicon esculentum	227	−	TTTTA	Core promoter element around −30 of transcription start
	Arabidopsis thaliana	129	−	TATAA	Core promoter element around −30 of transcription start
	Arabidopsis thaliana	225	+	TATAAA	Core promoter element around −30 of transcription start
	Lycopersicon esculentum	197	+	TTTTA	Core promoter element around −30 of transcription start
	Brassica napus	128	+	ATTATA	Core promoter element around −30 of transcription start
	Arabidopsis thaliana	130	−	TATA	Core promoter element around −30 of transcription start
CAAT-box	Arabidopsis thaliana	2	+	CCAAT	Common *cis-*acting element in promoter and enhancer regions
	Brassica rapa	232	+	CAAAT	Common *cis-*acting element in promoter and enhancer regions
	Arabidopsis thaliana	214	+	CCAAT	Common *cis-*acting element in promoter and enhancer regions
	Hordeum vulgare	3	+	CAAT	Common *cis-*acting element in promoter and enhancer regions
	Hordeum vulgare	215	+	CAAT	Common *cis-*acting element in promoter and enhancer regions
WUN-motif	Brassica oleracea	177	+	TCATTACGAA	Wound-responsive element
Chs-CMAla	Daucus carota	109	−	TTACTTAA	Part of a light responsive element
Box I	Pisum sativum	92	−	TTTCAAA	Light responsive element
ARE	Zea mays	153	−	TGGTTT	*Cis-*acting Regulatory element essential for the anaerobic induction
ATCT-motif	Arabidopsis thaliana	36	+	AATCTAATCT	Part of a conserved DNA module involved in light Respinsiveness
MBS	Arabidopsis thaliana	219	+	CAACTG	MYB binding site involved in drought-inducibility
Unnamed_1	Zea mays	101	+	CGTGG	
Unnamed_3	Zae mays	101	+	CGTGG	
Unnamed_4	Petroselinum hortense	190	+	CTCC	

Based on the sequence analysis results, anaerobic induction, wound induction, strong light induction, and drought induction experiments were performed on the T1 generation of transgenic tobacco. On the other hand, due to the presence of *cis-*acting elements of unknown function in WY195, temperature induction, which is very common in the induction type, has also been carried out. GUS activity was detected in plants subjected to high temperature and drought conditions (Figure 3E), and not detected in plants subjected under other induced conditions.

### WY195-Regulated Expression of *hpaXm* in Tobacco

**FIGURE 4:**
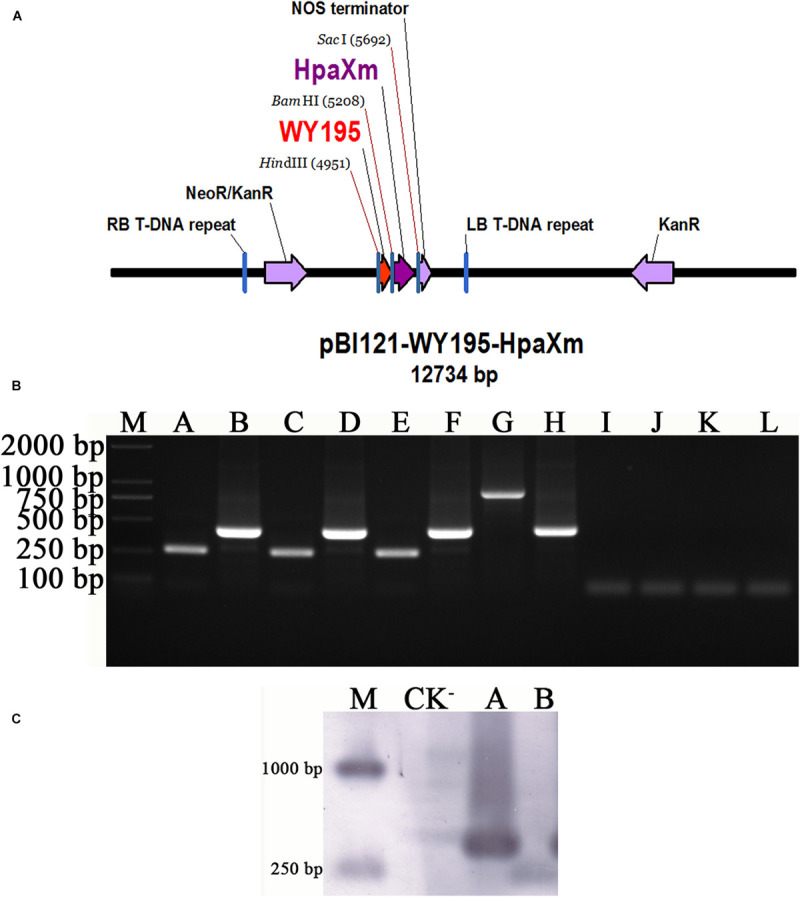
WY195-regulated expression of *hpaXm* in tobacco. **(A)** Map of the recombinant vector pBI121-WY195-*hpaXm.* **(B)** PCR amplification. (M) Marker 2000; (A) WY195-*hpaXm* transgenic tobacco plants, primers: WY195F/R; (B) WY195-*hpaXm* transgenic tobacco plants, primers: hpaXmF/R; (C) CK^+^, template: *O. heveae* wild type, primers: WY195F/R; (D) CK^+^, template: *X. citri*, primers wild type: hpaXmF/R; (E) CK^+^, template: recombinant vector pBI121-WY195-*hpaXm*, primers: WY195F/R; (F) CK^+^, template: recombinant vector pBI121-WY195-*hpaXm*, primers: hpaXmF/R; (G) CK^+^, template: 35S-*hpaXm* transgenic tobacco plants, primers: 35SF/R; (H) CK^+^, template: 35S-*hpaXm* transgenic tobacco plants, primers: hpaXmF/R; (I) CK^−^, template: *N. tabacum* wild type, primers: WY195F/R; (J) CK^−^, template: *N. tabacum* wild type, primers: hpaXmF/R; (K) CK^−^, template: ddH_2_O, primers: WY195F/R; (L) CK^−^, template: ddH_2_O, primers: hpaXmF/R. **(C)** PCR-Southern blot analysis. (M) DNA molecular weight marker (DIG-labeled); (CK^−^) *N. tabacum* wild type; (A) WY195 promoter; (B) *hpaXm*.

The plant expression vector pBI121-WY195-*hpaXm* was constructed (Figure 4A) and then transformed into *N. tabacum* using the ATMT method. PCR and Southern blot demonstrated that WY195 and *hpaXm* were successfully transformed the T1 generation transgenic tobacco plants (Figures 4B,C). The WY195 primer amplified a band of the same size as WY195, which was about 250 bp. The *hpaXm* primer was amplified with *hpaXm*, generating the expected band of approximately 400 bp (Figure 4B). The integration of the target genes was confirmed by Southern blot analysis (Figure 4C). PCR products amplified in the transgenic T1 generation were hybridized with corresponding probes targeting WY195 and *hpaXm* to produce two unique bands of the corresponding size. This indicated that WY195 and *hpaXm* genes had been successfully integrated into the genome of *N. tabacum*.

#### TMV Resistance

**FIGURE 5:**
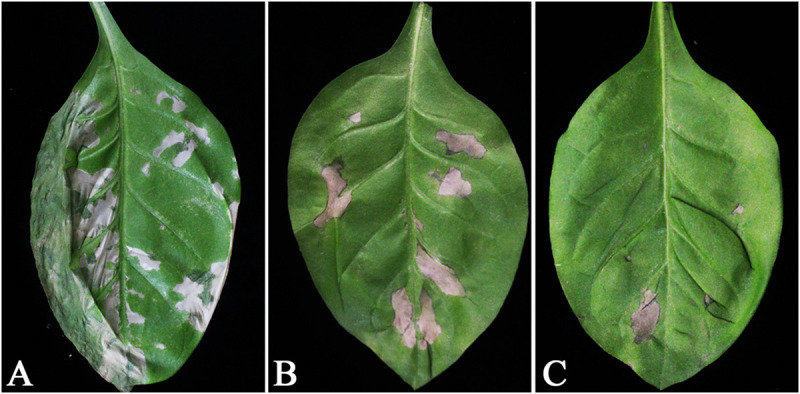
Leaves of transgenic *N. tabacum* plants inoculated with TMV (1.8 μg). Leaves transformed with **(A)** *hpaXm* or **(B)** vector pBI121, and **(C)** wild type. Lesions are present on leaves susceptible to TMV. Similar results were obtained from five sets of experiments: a total of 30 plants of each genotype were inoculated.

First, the T1 generation WY195-hpaXm transgenic tobacc is induced correspondly, then challenged with TMV rub inoculation. As a result, *hpaXm* transgenic tobacco showed significant TMV resistance compared to *N. tabacum* wild type (Figure 5). The number of TMV lesions observed on *N. tabacum* wild type after inoculation with TMV was significantly higher than that of the positive control 35S-*hpaXm* and WY195-*hpaXm* transgenic T1 plants (*P* ≤ 0.01; Table 3). Compared with *N. tabacum* wild type, the average number of lesions observed on the positive control group was approximately 29.37% lower. Compared with wild-type tobacco and the positive control, the average number of lesions observed on the WY195-*hpaXm* transgenic T1 plants was approximately 66.44 and 52.48% lower, respectively.

**TABLE 3:** Resistance levels of WY195-*hpaXm* transgenic tabacco against TMV.

**Plants**	**Plant leaf lesion number (X ± SE)**	***P* ≤ 0.01 Significance level^*a*^**	**Lesion reduction/%**
*N. tabacum* wild type	47.67 ± 1.43	A	
T1 generation of 35S-*hpaXm* transgenic tobacco	33.67 ± 2.33	B	29.37
T1 generation of WY195-*hpaXm* transgenic tobacco	16.00 ± 2.31	C	66.44

*SE, standard error. ^*a*^P ≤ 0.01 Significance level, Different letters indicate statistically significant differences between treatments according to Duncan’s multiple range test at P ≤ 0.01.*

#### Soluble Proteins Produced by WY195-*hpaXm* Transgenic Tobacco Elicited HR on Tobacco Leaves

**FIGURE 6:**
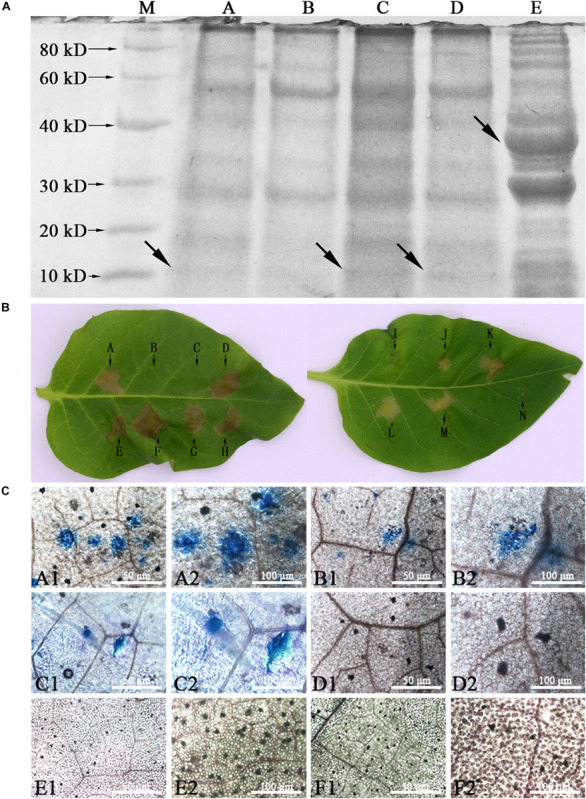
Analysis of soluble proteins from WY195-*hpaXm* transgenic tobacco. **(A)** Sodium dodecyl sulfate-polyacrylamide gel electrophoresis (PAGE) patterns of soluble protein preparations. (A) Soluble protein preparations (1 μL; 308 ng) including hpaXm from induced T1 generation WY195-*hpaXm* transgenic tobacco. (B) Soluble protein preparations (1 μL; 361 ng) from *N. tabacum* wild type; (C) Soluble protein preparations (1 μL; 366.5 ng) from pBI121-*hpaXm* transgenic plant; (D) Soluble protein preparations (1 μL; 301 ng) including hpaXm from uninduced T1 generation WY195-*hpaXm* transgenic tobacco. (E) Soluble protein preparations (1 μL; 325 ng) including hpaXm associated with a GST mark. The arrows represent the harpins HpaXm and GST-HpaXm (E). **(B)** Soluble proteins of hpaXm from WY195-*hpaXm* transgenic tobacco could stimulate HR. Bioactivity assays of proteins from leaf tissues, compared with the preparations of GST-hpaXm from *E. coli*. (A) GST-hpaXm (325 ng/μL) (CK^+^); (B) *N. tabacum* wild type (CK^−^) (361 ng/μL); (C) pBI121 transgenic tobacco (344 ng/μL); (D), (E), and (F) induced T1 generation WY195-*hpaX*m tobacco (308 ng/μL); (G) and (H) pBI121-*hpaXm* transgenic tobacco (366.5 ng/μL) (CK^+^); (I) PBS buffer (1 μL); (J), (K), (L) and (M) uninduced WY195-*hpaXm* transgenic tobacco (288 ng/μL); (N) ddH_2_O (CK^–^). **(C)** Trypan blue staining of WY195-*hpaXm* transgenic tobacco leaves. Micro-HRs were shown as areas stained blue. (A1) and (A2) induced WY195-*hpaXm* transgenic *N. tabacum*; (B1) and (B2) pBI121-*hpaXm* transgenic *N. tabacum*; (CK^+^); (C1) and (C2) with exogenous application of the hpaXm proteins (CK^+^); (D1) and (D2) pBI121 transgenic *N. tabacum* (CK^−^); (E1) and (E2) uninduced WY195-*hpaXm* transgenic *N. tabacum*; (F1) and (F2) *N. tabacum* wild type (CK^−^).

Sodium dodecyl sulfate–polyacrylamide gel electrophoresis (SDS-PAGE) gel patterns and bioactivity of soluble proteins from leaves of WY195-*hpaXm* transgenic tobacco, induced WY195-*hpaXm* transgenic tobacco, pBI121-*hpaXm* transgenic tobacco and *N. tabacum* wild type were compared. The molecular mass of hpaXm was estimated to be 13.3 kDa, and the putative GST–hpaXm protein was about 35 kDa (Miao, 2009). The results suggested that the purified protein from the induced WY195-*hpaXm* transgenic tobacco was abundant among total proteins and had the same size as hpaXm from *E. coli* (Figure 6A). Same size protein as hpaXm were not obtained for uninduced WY195-*hpaXm* transgenic lines. Only proteins from induced WY195-*hpaXm* transgenic tobacco and pBI121-*hpaXm* transgenic tobacco were active, and the degree of protein-induced HR from the induced WY195-*hpaXm* transgenic tobacco was significantly stronger than that of the protein from pBI121-*hpaXm* transgenic tobacco and hpaXm prepared periodically from recombinant *E. coli* strain (Figure 6B), even though the proteins were boiled for 10 min. Instead, proteins isolated from uninduced WY195-*hpaXm* transgenic tobacco, pBI121 transgenic tobacco or *N. tabacum* wild type did not cause the HR (Figure 6B). It demonstrated that hpaXm was actively present in leaf tissues of induced WY195-hpaXm transgenic tobacco.

#### Defensive Responses Were Induced in WY195-*hpaXm* Transgenic Tobacco

The T1 generation WY195-*hpaXm* transgenic tobacco was observed to determine whether defensive responses had been induced. As a result, no visible HR or micro-HR was induced on its leaf surface. However, dark blue micro-HR of scattered necrotic cell clusters were observed under the microscope after trypan blue staining, which was significantly more severe than pBI121-*hpaXm* transgenic tobacoo (positive control). Micro-HR was not induced in the *N. tabacum* wild type (negative control) (Figure 6C). The results showed that the expression of *hpaXm* which was regulated by WY195 promoter produced defensive responses with partial hypersensitive cell death in transgenic tobacco leaves, and it is more severe than that in pBI121-*hpaXm* transgenic tobacco.

### Preliminary Analysis of the Full Length of WY195 by a Series of Deletion Mutations

**FIGURE 7:**
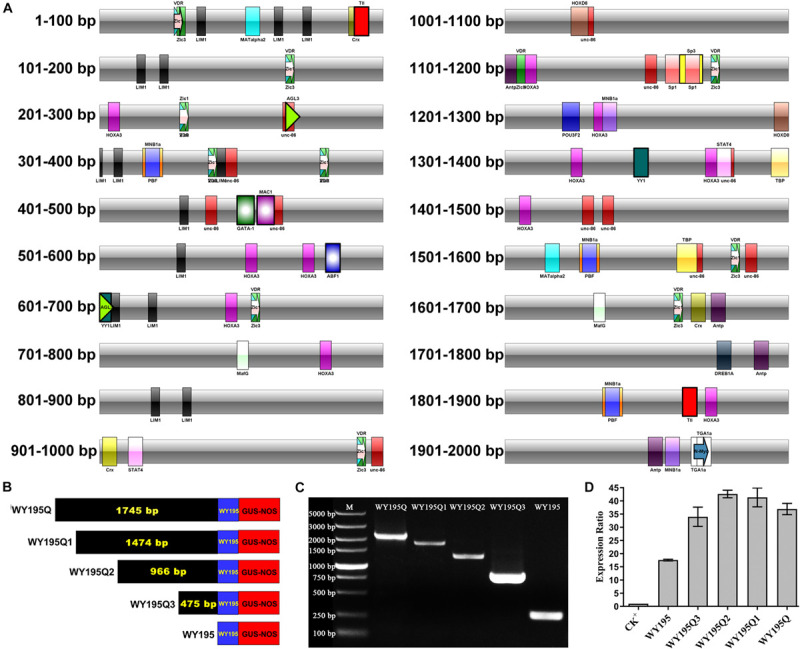
Preliminary analysis of the full length of WY195 by a series of deletion mutations. **(A)** Detailed distribution of transcription factor binding sites on the WY195Q sequence. **(B)** Schematic diagram of fusion of the 5′ series deletion fragment of WY195Q with the GUS gene. **(C)** PCR verification of the WY195 promoter series deletion mutant vector. **(D)** WY195Q series deletion mutant fragment drives the transient expression of *GUS* gene in *N. tabacum.*

In order to maximize the accuracy of the prediction, we reduced the error tolerance rate of PROMO to 0% and 103 TFs were obtained for WY195Q. The 103 TFs were searched in the online non-redundant database JASPAR of the TFBS, and all databases of all species were selected. A total of 27 previously reported TFBS were retrieved. These 27 TFBS with known functions were marked on the WY195Q sequence (Figure 7A). Serial deletion mutations of WY195Q were performed after avoiding the destruction of these TFBS. The plant expression vector pBI121-WY195Q, pBI121-WY195Q1, pBI121-WY195Q2 and pBI121-WY195Q3 were constructed for sequence WY195Q (2,100 bp), WY195Q1 (1,829 bp), WY195Q2 (1,321 bp), and WY195Q3 (830 bp) (Table 4 and Figures 7B,C). The level of transient expression of the GUS gene regulated by these sequences in tobacco was measured by qRT-PCR (Figure 7D). Among them, WY195Q2 regulates the highest expression of GUS gene. Thus the WY195 full length was preliminary analyzed as 1,321 bp.

**TABLE 4:** Primers for WY195Q series deletion mutation.

**Fragments**	**Upstream primers**	**Downstream primers**
WY195Q	195QF: 5′-CCCAAGCTTTGTATCTCGAGATCGTTTTTT-3′	5′-CGCGGATCCTCTGCATGCTAGTGATTTGT-3′
WY195Q1	195Q1F: 5′-CCCAAGCTTTTCTTCACCTAATTTTCGTCAGTCC-3′	
WY195Q2	195Q2F: 5′-CCCAAGCTTCCAGGCTTAGAATATTATTTATG-3′	
WY195Q3	195Q3F: 5′-CCCAAGCTTTCTAAGACGACATGTAATTATCC-3′	
WY195	195F: 5′-CCCAAGCTTAACCAATAATTTTCACGAGGG-3′	

## Discussion

Filamentous fungi has great potential promoter resource (Gouka et al., 1997), but up to now, there have been fewer promoters of filamentous fungi that have been developed. Most of the filamentous fungal endogenous promoters that have been applied or developed are used to express endogenous genes or to express exogenous genes in their own, but rarely used to express exogenous genes in plants or animals. Although it has greatly developed the endogenous genes of filamentous fungi, the application of these excellent promoters is relatively limited. Among the filamentous fungal promoters, there are likely to be excellent promoters that can applied to various organisms like the 35S promoter. In addition, filamentous fungal promoters that have been developed have relatively narrow sources in filamentous fungi. Due to the characteristics of obligate parasitism, the molecular research of obligate parasitic fungi is very backward, and its promoter has not been reported. The WY195 promoter investigated in this study was derived from *O. heveae*. *O. heveae* is an obligate parasitic fungus, it obviously is a very big surprise discovery.

Comparative genomics studies have shown that eukaryotic promoter sequences are more complex than prokaryotic promoter sequences, and that the mechanism of transcriptional regulation is more complicated (Kent, 2002). In eukaryotes, the transcriptional regulation mechanisms of plants are more complex than those of animals and the study of plant promoters is currently lagging behind that of animal studies (Li et al., 2000; Kent, 2002). In plant genetic engineering, microorganisms and plants are two main sources of promoters that used for regulating constitutive expression of genes. Virus-derived ones such as the 35S promoter and the FMV 34S promoter (Graff et al., 2003; Chi-Ham et al., 2012), plant-derived ones such as rice ACT1 and maize Ubi1 promoter, and the like. However, there are very few promoters that can efficiently express exogenous genes at a high level in both monocotyledons and dicotyledons. The constitutive promoter *Cauliflower mosaic virus* 35S (CaMV 35S) was first reported in 1985 (Odell et al., 1985). Since then, the 35S promoter has become the most widely and frequently used promoter in plant biotechnology. Almost every genetically modified crop plant that is grown commercially carries a version of this promoter (Hull et al., 2000). Even so, the 35S promoter is relatively less used in monocots (Wei, 2014) because the expression of exogenous genes regulated by the 35S promoter in monocots is much lower than that in dicots (expression has been reported to be up to 100-fold lower (Gallo-Meagher and Irvine, 1993). The rice Actin1 (ACT1) and the maize Ubiquitin1 (Ubi1) promoters are widely used in monocots, which are tens or even hundreds of times more efficient at regulating the expression of exogenous genes than the 35S promoter (Mcelroy et al., 1990; Last et al., 1991; McElroy et al., 1991; Christensen et al., 1992; Gallo-Meagher and Irvine, 1993). By contrast, in dicotyledons, the expression of exogenous genes regulated by the rice ACT1 or maize Ubi1 promoter is weak (Mcelroy et al., 1990).

Our results demonstrate that WY195 is a very efficient promoter and that its ability to drive the expression of exogenous genes in *N. tabacum* is far superior to that of the 35S promoter (17.54 times). We cannot only use WY195 to express its own endogenous gene in *O. heveae*, but also use WY195 to express a very useful exogenous gene in *O. heveae*. More importantly, WY195 can be used to express useful exogenous genes in plants, providing a new tool and method for plant genetic engineering. It has very good application potential. Further research indicates that WY195 has a very wide range of transcriptional regulation. WY195 can also express the exogenous gene *GUS* in monocotyledon rice. And its ability to regulate *GUS* expression is also very good, 5.09 times of the ACT1 promoter. At the same time, the results of transient expression indicated that WY195 can also express the exogenous gene *GUS* well in dicotyledonous rubber and dragon fruit, as well as monocotyledonous barley and maize. WY195 has the advantage of a wide range of transcriptional regulation, making it a much broader application and value. The efficient regulation of the transient expression of exogenous genes in rubber by the WY195 promoter is particularly encouraging given that the obligate biotrophic nature of *O. heveae* has hampered research studies on this pathogen and, therefore, our understanding of the molecular basis of its pathogenesis is still limited.

Over time, the deficiencies and defects of constitutive promoters have gradually emerged (Gittins et al., 2000). If exogenous genes are expressed in whole plants, a large amount of heterologous proteins or metabolites will accumulate in plants, upsetting the original metabolic balance of plants. In addition, some products may be toxic, hinder the normal growth of plants, and even lead to plant death (Lessard et al., 2002). By contrast, organ/tissue-specific promoters and inducible promoters can regulate gene expression more precisely in terms of time and space, thus making up for the deficiencies of constitutive promoters. Another advantage of developing organ/tissue-specific promoters and inducible promoters is that they are perceived to be a way of improving the safety of genetically modified foods. In the transgenic crops, the organ/tissue-specific promoters can be used to control the expression of the exogenous genes in specific non-edible organs. The inducible promoters can be used to control the expression of exogenous genes only under specific induction conditions, but not in a normal growth environment or in a harvested environment. This will prevent the presence of exogenous proteins in our food, such increasing the safety of genetically modified foods.

Given that *GUS* staining was not observed in the tissues or organs of the T1 generation of transgenic tobacco plants, we concluded that WY195 is not a constitutive promoter or an organ/tissue-specific promoter but an inducible promoter. Given that GUS activity was detected in tissues of plants subjected to high temperature and drought conditions, we inferred that WY195 is a high-temperature and drought-inducible promoter. We have confirmed that WY195 can regulate the expression of exogenous genes in major crops such as rice, corn and barley. In crops, if WY195 is used rationally to regulate the expression of beneficial exogenous genes such as yield increase and disease resistance, the expression of these genes is stimulated by the induction factors such as high temperature and drought during the growing season of crops, and the stimulation of these factors is avoided during the harvest period of agricultural products. It can increase the yield and quality of crops, and can also reduce the safety risks of genetically modified foods as much as possible. The series of deletion mutations is only a preliminary analysis of the full length of the WY195 promoter. In the next work, we will perform functional verification on WY195’s unreported TFs, and then analyze the exact full length of WY195.

Lots of reports have confirmed that exogenous application of harpins can enhance the systemic disease resistance of plants, such as tobacco, cucumber, rice, *Arabidopsis* and so on (Strobel et al., 1996; Ren, 2007; Chen et al., 2008a,b; Li et al., 2017). Similarly, *hrp* gene transgenic plants showed resistance to plants (Shao et al., 2008; Choi et al., 2012; Li et al., 2017). Results in our study supported the view, hpaXm also induced resistance when acting both intercellular and intracellular. HpaXm could confer defense responses without HR cell death against diverse plant pathogens when using exogenous application. *HpaXm* expression regulated by WY195 promoter can enhanced more resistance of transgenic tobacco than that regulated by 35S to TMV. There was no visible necrotic spots induced in WY195-*hpaXm* transgenic tobacco. This should be because WY195 is an inducible promoter. The *hpaXm* expression was very little or absent when the corresponding induction was not performed. When the corresponding induction was carried out, we observed a large amount of micro-HR on the transgenic tobacco leaves after trypan blue staining. This demonstrated that endogenous expression of *hpaXm* induced defensive responses in plants with cell death.

In summary, we have predicted, screened, function verified and developed a new endogenous inducible promoter of *O. heveae*, which was named WY195. WY195 has driven high levels of *GUS* expression in monocotyledonous and dicotyledons. *GUS* expression regulated by the WY195 promoter was 17.54-fold greater than that obtained using the 35S promoter in dicotyledons (*N. tabacum*) and 5.09-fold higher than that obtained using the ACT1 promoter in monocotyledons (*O. sativa*). Furthermore, WY195-regulated *GUS* gene expression was induced under high-temperature and drought conditions. WY195 can regulate the *hpaXm* expression in tobacco, and the generated protein hpaXm induced the micro-HR. Disease resistance bioassays showed that WY195-*hpaXm* transgenic tobacco enhanced the resistance to TMV. On the one hand, WY195 can provide new methods and tools for genetic engineering. On the other hand, our research and further in-depth research in the future will help to better understand the transcriptional regulation mechanisms and the pathogenic mechanisms of *O. heveae*.

## Data Availability Statement

The datasets presented in this study can be found in online repositories. The names of the repository/repositories and accession number(s) can be found below: https://www.ncbi.nlm.nih.gov/genbank/, MK049253.

## Author Contributions

YW and WM: conceptualization and formal analysis. YW: data curation and writing—original draft. WM and FZ: funding acquisition and supervision. YW, CW, LZ, and XX: investigation. YW, MR, and WL: visualization. YW, WM, and MR: writing—review and editing. All authors contributed to the article and approved the submitted version.

## Conflict of Interest

The authors declare that the research was conducted in the absence of any commercial or financial relationships that could be construed as a potential conflict of interest.

## Footnotes

1 http://www-bimas.cit.nih.gov/molbio/proscan/

2 http://alggen.lsi.upc.es/cgi-bin/promo_v3/promo/

3 http://jaspar.genereg.net/
